# Burden of multiple sclerosis in Iran from 1990 to 2017

**DOI:** 10.1186/s12883-021-02431-1

**Published:** 2021-10-15

**Authors:** Nima Fattahi, Sahar Saeedi Moghaddam, Farnam Mohebi, Negar Rezaei, Masoud Masinaei, Sahar Mohammadi Fateh, Elham Soleymani Hassanlouei, Farhad Manoochehri, Eghbal Fattahi, Mohammad Ali Sahraian, Maziar Moradi-lakeh, Ali H. Mokdad, Mohsen Naghavi, Farshad Farzadfar

**Affiliations:** 1 Non-Communicable Diseases Research Center, Endocrinology and Metabolism Population Sciences Institute, Tehran University of Medical Sciences, Tehran, Iran; 2 Endocrinology and Metabolism Research Center, Endocrinology and Metabolism Clinical Sciences Institute, Tehran University of Medical Sciences, Tehran, Iran; 3 Department of Epidemiology and Biostatistics, Tehran University of Medical Sciences, Tehran, Iran; 4 Student Research Committee, Kurdistan University of Medical Sciences, Sanandaj, Iran; 5 Department of Internal Medicine, Tohid Hospital, Kurdistan University of Medical Sciences, Sanandaj, Iran; 6 Department of Neurology, Sina Hospital, Tehran University of Medical Sciences, Tehran, Iran; 7 Preventive Medicine and Public Health Research Center, Iran University of Medical Sciences, Tehran, Iran; 8 Institute for Health Metrics and Evaluation, University of Washington, Seattle, WA USA

**Keywords:** MS (multiple sclerosis), Burden of disease, Iran, Global burden of disease study, Trend

## Abstract

**Background:**

Multiple Sclerosis (MS) is a burdensome, chronic and autoimmune disease of the central nervous system. We aimed to report the incidence, prevalence, mortality, and Disability Adjusted Life Years (DALYs) of MS in Iran at a national level for different age and sex groups over a period of 28 years (1990–2017).

**Methods:**

Data were extracted from the Global Burden of Disease study (GBD) from 1990 to 2017, published by the Institute for Health Metrics and Evaluation. The incidence of DALYs and prevalence of MS were estimated to report the burden of MS based on sex and age in Iran from 1990 to 2017.

**Results:**

At the national level, the Age-Standardized Incidence Rate (ASIR), Age-Standardized Prevalence Rate (ASPR), Age-Standardized DALYs Rate (ASDR) and the Age-Standardized Mortality Rate (ASMR) in Iran in 2017 were 2.4 (95% Uncertainty Interval [UI]: 2.1 to 2.7), 69.5 (62.1 to 77.8), 29.1 (23.6 to 34.7), and 0.4 (0.3 to 0.4) per 100,000 population, respectively. During the period of 1990 to 2017, all measures increased, and were higher among females. The incidence rate began upward trend at the age of 20 and attained its highest level at the age of 25.

**Conclusion:**

In Iran, all of the age-standardized MS rates have been increasing during the 28 years from 1990 to 2017. Our findings can help policy makers and health planners to design and communicate their plans and to have a better resource allocation, depending on the incidence and prevalence of the growing numbers of MS patients in Iran.

## Background

Multiple Sclerosis (MS) is a central nervous system disease that is categorized as an autoimmune and chronic disease that causes nerve damage, inflammation and demyelination of the nerves [1–5]. The disease irreversibly destroys axons and myelin to varying degrees [5, 6]. MS was considered as a T-cell mediated disease with an abnormal balance between regulatory T-cells and Central Nervous System (CNS) reactive effector T-cells. Recently added evidence shifted attention to B cells’ potential contributions on CNS inflammatory disease activity, mainly the antibody-independent functions of B cells implicated in mediating new relapsing MS disease activity as part of cascades of cellular immunological interactions in the periphery [7]. The main cause of the illness is not clearly known, but a few genetic and environmental factors are involved in the onset of the disease, which include positive serology of the nuclear antigen of the Epstein bar virus, infectious mononucleosis and smoking [3]. Other possible causes of the disease include lack of vitamin D intake, genetic risk factors [6], geographical distribution, so that the studies have shown that MS prevalence and incidence increases with latitude gradient [3, 8]. Regarding diet, hypercaloric Western-style diets, characterized by high salt, animal fat, red meat, sugar-sweetened drinks, fried food, low fiber, and lack of physical exercise are other predisposing factors of MS [9].

Based on the MS International Federation report, the global mean prevalence rate of MS increased from 30 cases per 100,000 population in 2008 to 33 cases per 100,000 population in 2013. The lowest rates of 2 (1.71 to 2.29) and 2.8 (2.4 to 3.1) were seen in Oceania and Central Sub-Saharan Africa, respectively. Conversely, the highest prevalence rates were seen in Nova Scotia )266.9(and British Columbia) 179.9(of Canada, and North America )164.6) [1, 4]. Since MS does not lead to quick deaths, reporting other measures in addition to the incidence and prevalence can help better measure the burden of the disease [10]. In this regard, the Disability Adjusted Life Year (DALY) is used as one of the most applicable measures for evaluating community health. In 2013, globally, a total of 1,343,000 DALYs and 1920 ASDR per 100,000 people were reported for all age groups affected with MS. Moreover, 1.6% of all reported neurological diseases’ DALYs were MS related [6].

With a mean prevalence of 51.52 cases per 100,000 population, Iran is categorized as a high prevalence region in the middle east [11]. Some studies in central areas of Iran showed that the MS prevalence rate in Tehran and Isfahan were 101 [11] and 85 cases per 100,000 population, respectively [12]. The increasing burden of MS, plus the high cost of managing this disease and the need to be able to track the change in incidence and prevalence through better data suggests that policymakers and health planners need accurate epidemiological information about its condition in the country [12]. In this study, as a part of the GBD study, we aimed to report the incidence, prevalence, mortality, and DALYs of MS in Iran at a national level in different sex and age groups over a period of 28 years (1990–2017).

## Methods

The components of data collection, statistical methods, and estimation processes for the GBD 2017 study have been described comprehensively elsewhere [13–15], where the burden of 354 diseases and injuries in 195 countries and territories by sex and age groups in terms of incidence, prevalence, death, DALYs, Years of Life Lost (YLL), and Years Lived with Disability (YLD) measures have been reported.

In this paper, we report the burden of MS in Iran from 1990 to 2017 by sex and age groups. International Classification of Diseases (ICD)-10 codes of G35-G35.0 and G35-G35.9 were mapped to define non-fatality and fatality of MS, respectively [14, 16]. The original data estimated by the GBD for MS in Iran was mostly from death registry system, MS registry and scientific literatures. Rates are expressed as age-standardized based on the GBD reference population [17]. 95% Uncertainty Intervals [18] were calculated with the 2.5th and 97.5th percentiles of 1000 draws by age, sex, location, and year, and point estimates were calculated from the median estimates across the draws for each measure.

Decomposition analysis was applied between 1990 and 2017 to determine the contribution of change in the age-specific incidence rate, population growth, and population aging of new cases [19]. Percent changes for number and rate were calculated as the difference between the last and first study years divided by each measure’s initial value. We computed expected burden on the basis of sociodemographic index (SDI) and compared these estimates to observed rates. The SDI is calculated from the geometric mean of total fertility rate under 25 years, lag-distributed income per capita, and average educational attainment in the population older than 15 years [15]. The Mortality-to-Incidence Ratio (MIR) was calculated to measure quality of care and evaluate disease management outcome. Figures were depicted by R version 3.4.2.

## Results

**Fig. 1:**
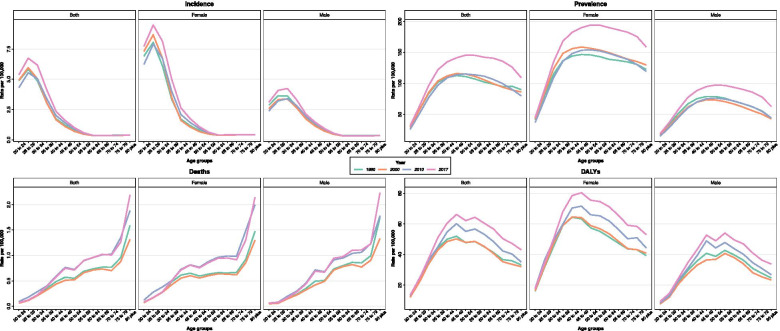
Age trend of age-standardized rates vs all age groups by measure from 1990 to 2017

**Fig. 2:**
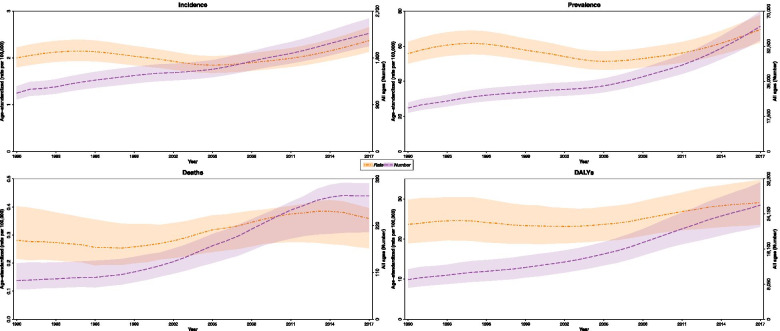
Time trend of age-standardized rates vs all age groups by measure from 1990 to 2017

**Fig. 3:**
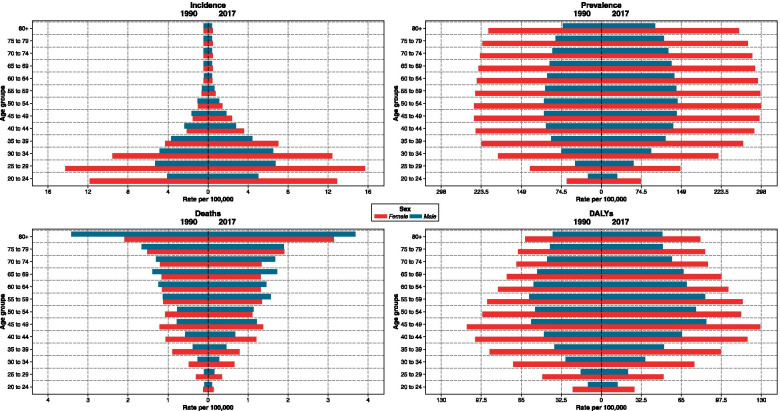
Comparison of each measure’s rate by age group and sex, 1990 vs 2017

At the national level, MS related Age Standardized Incidence Rate (ASIR) was 2.0 (95% UI: 1.8 to 2.2) and 2.4 (2.1 to 2.7) per 100,000 population in 1990 and 2017, respectively, with an average annual percentage change of 0.6. The females to male’s ratio of ASIR was 1.8 in 1990 and 1.9 in 2017 (Table 1). The age trend of the disease had been shown in the 1990s, 2000s, 2010 and 2017, and followed a similar pattern throughout all these years. The incidence rate began its upward trend from the age of 20 years and peaked at the age of 25. From the age of 25, a downward trend began and reached its lowest level at the ages of 55 to 59 (Fig. 1). The evaluation of the ASIR time trend showed that there had been a slight slope in the period from 1990 to 1995, after which it slightly decreased from 1996 to 2005 and then began to rise from 2005 until 2017 (Fig. 2). The population age pyramid of incidence rate in 1990 and 2017 indicated that the highest incidence rate was in the 25–29 years age group and then followed a downward trend (Fig. 3). Furthermore, decomposition analysis showed that 42.0% of the changes in the disease’s incidence cases were due to population growth, 26.2% due to the age structure changes and 34.1% due to changes in the incidence rate of the disease (Table 2).

**Table 1:** Burden of Multiple Sclerosis in 1990 and 2017 with percentage change by sex and location

Measure	Unit	Location	Both			Male			Female		
			1990	2017	Percent change ^**c**^	1990	2017	Percent change ^**c**^	1990	2017	Percent change ^**c**^
**Incidence**	**Number** ^**a**^	Global	40,417 (36,731 to 44,787)	54,895 (50,054 to 60,812)	35.8 (33.9 to 37.9)	14,996 (13,560 to 16,691)	19,930 (18,068 to 22,184)	32.9 (30.8 to 35.2)	25,421 (23,138 to 28,110)	34,964 (31,830 to 38,686)	37.5 (35.4 to 39.9)
		North Africa and Middle East	3674 (3240 to 4135)	7740 (6950 to 8646)	110.7 (102.5 to 120.3)	1295 (1133 to 1467)	2713 (2420 to 3049)	109.4 (100.8 to 119.2)	2379 (2102 to 2681)	5026 (4520 to 5600)	111.3 (102.5 to 121.6)
		Iran	1122 (1004 to 1254)	2271 (2024 to 2557)	102.3 (90.3 to 117.1)	390 (347 to 436)	794 (705 to 894)	103.7 (91.5 to 117.8)	732 (657 to 821)	1477 (1315 to 1666)	101.6 (89.3 to 117.3)
	**Rate** ^**b**^	Global	0.7 (0.7 to 0.8)	0.7 (0.6 to 0.8)	−6.1 (−7.1 to −5.1)	0.6 (0.5 to 0.6)	0.5 (0.5 to 0.6)	−10.8 (− 11.8 to − 9.7)	0.93 (0.85 to 1.03)	0.90 (0.82 to 1.0)	−3.3 (− 4.5 to − 2.0)
		North Africa and Middle East	1.1 (1.0 to 1.2)	1.2 (1.1 to 1.3)	7.9 (5.5 to 10.4)	0.8 (0.7 to 0.9)	0.8 (0.7 to 0.9)	1.2 (−1.1 to 3.8)	1.4 (1.3 to 1.6)	1.6 (1.5 to 1.8)	12.7 (9.8 to 15.7)
		Iran	2.0 (1.8 to 2.2)	2.4 (2.1 to 2.7)	18.5 (16.7 to 20.5)	1.4 (1.3 to 1.6)	1.6 (1.5 to 1.8)	12.3 (10.8 to 14.1)	2.6 (2.3 to 2.9)	3.1 (2.8 to 3.5)	22.5 (19.9 to 25.3)
**Prevalence**	**Number** ^**a**^	Global	1,048,261 (946,018 to 1,164,731)	1,761,078 (1,598,226 to 1,947,909)	68.0 (65.5 to 70.6)	335,891 (300,256 to 375,033)	551,872 (498,079 to 614,373)	64.3 (61.8 to 66.9)	712,369 (645,599 to 789,379)	1,209,206 (1,096,303 to 1,336,075)	69.7 (67.0 to 72.7)
		North Africa and Middle East	71,262 (62,756 to 80,648)	201,299 (181,311 to 223,278)	182.5 (174.6 to 191.9)	23,645 (20,552 to 26,789)	65,344 (58,438 to 72,850)	176.4 (168.2 to 185.8)	47,617 (41,965 to 53,835)	135,955 (122,509 to 150,603)	185.5 (176.7 to 195.2)
		Iran	21,662 (19,342 to 24,342)	62,397 (55,554 to 69,871)	188.1 (179.9 to 196.5)	7304 (6470 to 8182)	20,102 (17,828 to 22,498)	175.2 (167.7 to 183)	14,358 (12,810 to 16,143)	42,295 (37,748 to 47,790)	194.6 (185.1 to 204.6)
	**Rate** ^**b**^	Global	22.5 (20.3 to 24.9)	21.7 (19.7 to 24.0)	−3.4 (−4.6 to −2.1)	14.5 (13.0 to 16.2)	13.8 (12.4 to 15.3)	−5.0 (−6.1 to − 3.8)	30.1 (27.3 to 33.4)	29.3 (26.6 to 32.4)	−2.5 (− 4.0 to −1.0)
		North Africa and Middle East	28.9 (25.6 to 32.7)	35.8 (32.4 to 39.7)	23.9 (20.6 to 27.5)	18.7 (16.4 to 21.2)	22.2 (19.9 to 24.7)	18.3 (15.1 to 22.2)	39.6 (35.1 to 44.8)	50.5 (45.7 to 56.1)	27.6 (23.9 to 31.5)
		Iran	55.8 (50.1 to 62.5)	69.5 (62.1 to 77.8)	24.7 (22.7 to 26.9)	36.9 (32.8 to 41.5)	44.5 (39.7 to 49.8)	20.6 (18.7 to 22.7)	75.3 (67.6 to 84.5)	94.9 (85 to 107)	26.1 (23.4 to 29)
**Deaths**	**Number** ^**a**^	Global	12,290 (10,793 to 14,420)	20,655 (17,721 to 22,238)	68.1 (14.2 to 83.0)	5115 (4368 to 5869)	8452 (7123 to 9182)	65.2 (17.8 to 79.0)	7175 (6055 to 9009)	12,203 (10,082 to 13,629)	70.1 (12.0 to 87.5)
		North Africa and Middle East	371 (231 to 648)	1040 (706 to 1263)	180.0 (53.3 to 257.9)	170 (107 to 274)	489 (332 to 558)	187.7 (67.8 to 271.3)	202 (121 to 378)	551 (346 to 738)	173.5 (44.3 to 254.2)
		Iran	91 (71 to 131)	290 (205 to 319)	219.2 (61.1 to 305.8)	42 (31 to 58)	133 (89 to 148)	219.6 (75.8 to 301.5)	49 (37 to 74)	157 (111 to 176)	218.8 (52.5 to 323.4)
	**Rate** ^**b**^	Global	0.3 (0.3 to 0.3)	0.3 (0.2 to 0.3)	−10.1 (−38.8 to −2.3)	0.2 (0.2 to 0.3)	0.2 (0.2 to 0.2)	−11.7 (−37.2 to −4.8)	0.3 (0.3 to 0.4)	0.3 (0.2 to 0.3)	−9.2 (−39.8 to 0.0)
		North Africa and Middle East	0.2 (0.1 to 0.3)	0.2 (0.1 to 0.2)	16.2 (−35.8 to 46.7)	0.2 (0.1 to 0.3)	0.2 (0.1 to 0.2)	18.8 (−30.2 to 51.7)	0.2 (0.1 to 0.3)	0.2 (0.1 to 0.3)	14.8 (−39.2 to 47.6)
		Iran	0.3 (0.2 to 0.4)	0.4 (0.3 to 0.4)	27.4 (−34.3 to 60.3)	0.3 (0.2 to 0.4)	0.3 (0.2 to 0.4)	28.7 (−28.6 to 61.7)	0.3 (0.2 to 0.4)	0.4 (0.3 to 0.4)	26.9 (−38.1 to 66.1)
**DALYs**	**Number** ^**a**^	Global	682,944 (586,530 to 811,282)	1,084,757 (942,878 to 1,237,344)	58.8 (29.4 to 72.8)	262,002 (227,634 to 302,473)	407,529 (354,187 to 459,135)	55.5 (28.7 to 68.1)	420,942 (355,788 to 509,258)	677,228 (579,371 to 780,792)	60.9 (31.3 to 76.6)
		North Africa and Middle East	32,940 (24,924 to 46,413)	90,888 (71,930 to 110,100)	175.9 (111.4 to 210.6)	12,541 (9482 to 17,234)	34,807 (27,434 to 41,382)	177.5 (111.0 to 219.3)	20,399 (15,296 to 29,285)	56,082 (43,493 to 69,772)	174.9 (111.8 to 210.5)
		Iran	9098 (7244 to 11,463)	26,183 (21,205 to 31,381)	187.8 (122 to 222.9)	3420 (2752 to 4296)	9725 (7889 to 11,529)	184.3 (120.4 to 221.1)	5677 (4448 to 7296)	16,458 (13,232 to 19,728)	189.9 (124 to 229.2)
	**Rate** ^**b**^	Global	14.8 (12.8 to 17.5)	13.3 (11.5 to 15.2)	−10.2 (−26.8 to −2.7)	11.5 (10.0 to 13.2)	10.1 (8.8 to 11.4)	−11.9 (− 27.5 to −5.4)	18.0 (15.2 to 21.6)	16.3 (14.0 to 18.9)	−9.2 (− 25.6 to −0.6)
		North Africa and Middle East	13.6 (10.3 to 19.2)	16.1 (12.8 to 19.6)	18.3 (−9.9 to 33.2)	10.3 (7.8 to 14.2)	12.0 (9.5 to 14.2)	16.0 (−12.5 to 33.2)	17.1 (12.8 to 24.4)	20.6 (16.1 to 25.4)	20.7 (−7.3 to 35.9)
		Iran	23.7 (18.9 to 29.8)	29.1 (23.6 to 34.7)	23.1 (−5.1 to 37.1)	17.8 (14.4 to 22.5)	21.8 (17.6 to 25.7)	22.4 (−5.6 to 38.2)	29.7 (23.4 to 38.0)	36.6 (29.4 to 43.9)	23.4 (−3.9 to 38.9)

^a^ All ages; ^b^ Age-standardized rate per 100,000; ^c^ 1990–2017

**Table 2:** Decomposition analysis of factors affecting number of incident cases of Multiple Sclerosis during 1990 to 2017, Iran

Sex	% 1990–2017 incident case change cause			Overall change
	*Population growth*	*Age structure change*	*Incidence rate change*	
Both	42.0%	26.2%	34.1%	102.3%
Male	41.4%	38.2%	24.2%	103.8%
Female	42.7%	18.9%	40.0%	101.6%

In Iran, ASPR increased from 55.8 (50.1 to 62.5) per 100,000 people in 1990 to 69.5 (62.1 to 77.8) in 2017, with an average annual percentage change of 0.8 (Table 1). The ASDR due to MS in Iran increased from 23.7 (18.9 to 29.8) per 100,000 population in 1990 to 29.1 (23.6 to 34.7) in 2017, with an average annual percentage change of 0.7. The females to male’s ratio of ASDR was 1.6 in both 1990 and 2017 (Table 1). The evaluation of the DALYs rate age trend showed that an upward trend began at the age of 20 with a relatively steep slope and continued to peak until the age of 45. Afterwards, it declined by a slight downward slope and from the age of 55 it began to decline and continued so until death (Fig. 1). The MS related ASMR in Iran increased from 0.3 (0.2 to 0.4) per 100,000 population in 1990 to 0.4 (0.3 to 0.4) in 2017, with an average annual percentage change of 0.9 (Table 1). From the age of 20 to 55, mortality rate was always higher among females. However, the mortality rate in people over 55 years of age was higher among males (Fig. 3). ASMR and ASDR were respectively 1.3 and 1.7 times greater than the level expected in 2017 given the SDI level (Table 3).

**Table 3:** Observed and expected burden of Multiple Sclerosis in 1990 and 2017 by sex

Measure	Unit	Both						Male						Female					
		1990			2017			1990			2017			1990			2017		
		O	E	O/E ratio	O	E	O/E ratio	O	E	O/E ratio	O	E	O/E ratio	O	E	O/E ratio	O	E	O/E ratio
**Deaths**	**Number** ^**a**^	91	69	1.3	290	290	1.0	42	32	1.3	133	130	1.0	49	37	1.3	157	159	1.0
	**Rate** ^**b**^	0.3	0.2	1.5	0.4	0.3	1.3	0.3	0.2	1.5	0.3	0.3	1.0	0.3	0.2	1.5	0.4	0.4	1.0
**DALYs**	**Number** ^**a**^	9098	3621	2.5	26,183	14,947	1.8	3420	1485	2.3	9725	6121	1.6	5677	2116	2.7	16,458	8783	1.9
	**Rate** ^**b**^	23.7	7.9	3.0	29.1	16.8	1.7	17.8	6.7	2.7	21.8	13.8	1.6	29.7	9.2	3.2	36.6	19.8	1.8

^a^ All ages; ^b^ Age-standardized rate per 100,000

O: Observed; E: Expected

The ASIRs of MS attributable to smoking were 2.4 and 2.5 per 100,000 people in 1990 and 2017, respectively. Its male-to-female ratio was 3.0 in 1990 and 3.1 in 2017.

## Discussion

The results of this study showed that the age-standardized rates of all estimates for MS in 2017 were higher than in 1990. In addition, from the beginning to the end of the study all of the estimates were higher in women. Moreover, the highest incidence rate was among the 25–29 years age group while the highest DALY rate was among 45–49 year olds.

This study shows that the incidence and prevalence and DALY rates have increased in the last 10 years. A review study by Heydarpour et al. was conducted on population-based researches in the Middle East and North Africa. It indicated an upward trend in the prevalence of the disease in Tehran from 2008 to 2011 and also showed a rising incidence trend in Tehran in the last two decades. The ASIR of MS in Tehran increased from 0.68 in 1989 to 5.68 in 2005 [12]. In addition to Tehran, population-based studies in Isfahan revealed a general increase in the incidence and prevalence of the disease in the last decade, where ASPR increased from 35.5 per 100,000 people in 2006 to 85.8 in 2013 [12]. This finding was consistent with our study’s findings. On the other hand, a study by Mansouri et al. divided the whole world into seven major geographic regions and examined the incidence and prevalence of MS in these areas. Their results indicated that the incidence and prevalence trends of MS had decreased in 5 of the regions, which included Iran and the Middle East. Latin America and the Caribbean were the only 2 regions that showed increasing trends of incidence and prevalence in recent years. Lack of vitamin D intake and genetic risk factors have been cited as the possible causes of this increasing trend [6]. A longitudinal study from 2001 to 2013 evaluating the prevalence and trends of vitamin D deficiency in Iran showed that although the prevalence of vitamin D inadequacy (deficiency and insufficiency) decreased. The prevalence of vitamin D deficiency and insufficiency was still high in 2001, 2007, and 2013 (62.0, 57.9, and 53.9%). Mentioned rates of vitamin D inadequacy could be an essential cause of increasing trends of MS rates in Iran [20]. Since Heydarpour’s study reported the results of population-based studies, it has more reliable results compared to Mansouri’s study, which in order to estimate measures was based on modeling. This review study showed the highest ASPR to be in Tehran and Isfahan with 50.4 and 31.5 per 100,000 people, respectively [12]. A study by Eskandarieh et al. examined the status of MS in southern, eastern and Southeast Asia. It revealed that some Asian countries, including Iran and Japan, had reported an increasing prevalence of MS in the last decade. For instance, according to the Iranian Ministry of Health’s report, the prevalence of MS in 2011 in Iran was 45 per 100,000 people and 54.5 in 2013 [21]. In our study in 2017, this number had increased to 69.5 per 100,000 population.

One of the possible causes of this upward trend is the shift to a western lifestyle, smoking, UV-protection and low levels of vitamin D [12]. Other causes of the increasing incidence and prevalence trends of MS can be the unwanted side effects of increased health and the development of health facilities that have been developed under the heading of “hygiene theory”. The theory states that by increasing health, vaccination and the use of antibiotics in developed countries during the past decades, the immune system has changed improperly and people are now prone to autoimmune diseases [6]. In addition to the hygiene theory, the increasing number of diagnostic tools in recent years can lead to an increase in ASIR. The first MRI device arrived in Iran in 1990, after which the number of these devices slowly increased until 1995. And since 1999, the number of these devices has multiplied. In 2005 there were 93 devices in Iran, and the number of MRI devices per one million population was 1.93 in 2005 and 3.5 in 2016 [22, 23]. Furthermore, timely and advanced health care in developed countries can lead to an increase in the survival of patients and thus an increase in the ASPR of the disease [4, 6]. The Global Atlas of MS has shown that access to specialist services in low-income countries is 0.03 per 100,000 population and 3.6 in high-income countries. The number of Magnetic Resonance Imaging devices available in the world is 0.42 per 100,000 people. This number is 1.6 in high-income countries, 0.4 in lower-middle-income countries and 0.01 in low-income countries [24]. So, we can state that more diagnostic tools and facilities can increase the chance of MS detection.

In our study, the female-to-male ratios of ASIR and ASPR in 2017 were 1.9 and 2.1, respectively. This finding was in line with other studies conducted in Denmark, France, Australia and Norway, which also revealed an increase in the incidence and prevalence rates among females [3]. The searches conducted in the study by Tolou-ghamari et al., who examined the geo-epidemiological differences in MS in Iran and other Middle Eastern countries, showed that the epidemiology of this disease was changing and its incidence among the female population in recent years had doubled [25]. These findings are in line with our study’s findings. The environmental factors that are likely to contribute to the increased incidence of MS in women include increased cigarette smoking among women, changes in lactation patterns, increased obesity, increased gestational age, use of oral contraceptives, decreased physical activity, and increased stress [3, 4].

In our study, the 25–29 years age group had the highest incidence rates, and in the study by Eskandarieh et al., the highest prevalence rate was among 24–29 year-olds. These findings are consistent with our study’s findings [11]. The GBD 2017 study showed that the DALYs rate was highest in the sixth decade of life [15]; since it usually affects people from an early age and survival has also increased among these patients, leading to an increase in DALYs rate. In our study, ASDR reached its highest level in the late fifties, followed by a downward trend until the end of the patients’ lives. The DALY’s trend for MS has a different pattern from other neurological diseases. Although the incidence rate of MS is very low, it causes a lot of DALYs because it occurs at an early age [26]. New changes in the sex, race, and nationality of MS patients suggest the role of environmental factors in the development of this disease. The most important risk factors include infection with the Epstein bar virus, which starts from the stomach and spreads to the brain. Other risk factors include smoking, lack of sunlight, diet, obesity and gastric microorganisms. More than 200 alleles in the human genome have been identified as risk factors for MS and that genetics is a known risk factor for this disease [27].

This study had several strengths and limitations. One strength was that we used several databases including MS registry and death registry system’s data, then we used modeling methods for filling the gaps and calculating uncertainty, so we could cover the incompleteness of registry systems. One of the limitations of this study was the lack of categorization of results based on the different clinical courses of multiple sclerosis, which is due to the incompleteness of current registry systems. Based on clinical courses of the disease, MS is classified into four different types [28].

## Conclusion

In conclusion, the results of this study showed that during the study period, all MS age-standardized rates in Iran had been increasing. In addition, the sex ratio in all measures had always been higher among females. The geographical distribution of all MS measures also followed a specific pattern, with the highest rates observed in the north and northwestern regions of the country, and the lowest rates observed in the east, south and southeastern parts of the country. Our findings can help policymakers and health planners to design and communicate their plans and have a better resource allocation at provincial levels, depending on the incidence and prevalence of the growing numbers of MS patients in Iran.

## Data Availability

All data generated or analyzed during this study are publicly available dataset. MS data analyzed in this study are publicly available in the GBD data tool: (https://gbd2017.healthdata.org/gbd-compare).

## Abbreviations

MS: Multiple Sclerosis
DALYs: Disability Adjusted Life Years
GBD: Global Burden of Disease study
ASIR: Age-Standardized Incidence Rate
ASPR: Age-Standardized Prevalence Rate
ASDR: Age-Standardized DALYs Rate
ASMR: Age-Standardized Mortality Rate
CNS: Central Nervous System
YLLs: Years of Life Lost
YLDs: Years Lived with Disability
ICD: International Classification of Diseases
SDI: sociodemographic index
MIR: Mortality-to-Incidence Ratio
